# Immunomodulation Effect of Biomaterials on Bone Formation

**DOI:** 10.3390/jfb13030103

**Published:** 2022-07-25

**Authors:** Tong Zhao, Zhuangzhuang Chu, Jun Ma, Liping Ouyang

**Affiliations:** 1 Hongqiao International Institute of Medicine, Tongren Hospital, Shanghai Jiao Tong University School of Medicine, Shanghai 200336, China; tongzhao1218@163.com (T.Z.); czzlylotus@163.com (Z.C.); 2 Jiangsu Key Laboratory of Oral Diseases, Nanjing Medical University, Nanjing 210029, China; 3 Department of General Practitioners, Tongren Hospital, Shanghai Jiao Tong University School of Medicine, Shanghai 200336, China

**Keywords:** osteoimmunomodulation, bone regeneration, macrophages, biomaterials

## Abstract

Traditional bone replacement materials have been developed with the goal of directing the osteogenesis of osteoblastic cell lines toward differentiation and therefore achieving biomaterial-mediated osteogenesis, but the osteogenic effect has been disappointing. With advances in bone biology, it has been revealed that the local immune microenvironment has an important role in regulating the bone formation process. According to the bone immunology hypothesis, the immune system and the skeletal system are inextricably linked, with many cytokines and regulatory factors in common, and immune cells play an essential role in bone-related physiopathological processes. This review combines advances in bone immunology with biomaterial immunomodulatory properties to provide an overview of biomaterials-mediated immune responses to regulate bone regeneration, as well as methods to assess the bone immunomodulatory properties of bone biomaterials and how these strategies can be used for future bone tissue engineering applications.

## 1. Introduction

Bone loss in many situations, including aging, pathological fracture, periodontitis, and osteomyelitis, can lead to poor physical conditions, most of which require bone replenishment and timely surgical repair using implantation materials [1,2,3]. Nearly all dominant bone biomaterials have been developed and produced following the principle of great physicochemical properties and biocompatibility. Using this principle, candidate materials are usually subjected to the in vitro simulation of osteogenesis, which is intrinsically driven by osteoblastic activities in vivo [4,5]. In addition, owing to the advancement in material science, the fabrication of implants can meet the practical demands of patients.

Nevertheless, owing to the inconsistent outcomes of candidate materials in vitro and in vivo, it is not easy for these existing biomaterials to transform into clinically applicable implant materials in the human body. The reasons are that on the one hand, the physicochemical, biological, and mechanical properties of the candidate materials must certainly be optimized to meet specific demands in our complexed organic environment. On the other hand, as we obtain an increasingly profound understanding of bone biology and the underlying mechanisms of osteogenesis, we understand that the musculoskeletal system is not the sole contributor to this process. Osteogenesis is a composite bioprocess that collaborates with diverse molecular events. These concerns cause limitations in the dependence and efficiency of traditional bone biomaterials for practical purposes [6,7], and novel insights into the fabrication principle and operating mechanisms of bone biomaterials are urgently needed.

Osteoimmunology, an emerging theory derived from the latest findings in bone biology, hypothesizes that immune responses play essential roles in bone formation and homeostasis. Many functional proteins, signaling molecules, and cytokines have been confirmed to participate synchronously in reactive immune events and osteogenesis. Thus, immune signaling pathways are closely related to bone formation [8,9]. An immune response is defined as the body’s defense against foreign matter or mutated autologous components. Triggered by the recognition of antigens, the entire process can generate an immune response to an antigen, including immune induction by antigens to the body, interactions between immune cells, and multiple effects mediated by immune effectors (e.g., sensitized lymphocytes, antibodies) [10]. Naturally recognized as a foreign body in this environment, the implant could provoke a series of adverse and even fatal events derived from the immune system, which can determine the destiny of bone biomaterials. In this type of situation, the irrevocable damage caused by various immune effector cells significantly changes the physicochemical and biological properties that are meant to help with bone formation in vivo, and the unexpected disturbance makes it difficult for biomaterials to work successfully in vitro. For example, the inappropriate immune responses to foreign implants which elicit excessive inflammation could lead to the formation of a fibrous capsule. The immune-related alterations significantly weaken the osteogenesis capability of biomaterials by preventing them from contacting and coordinating with bone cells. To reverse the impact, Chen and colleagues attached an SZS coating to Ti–6Al–4V and endowed the implant with immunomodulatory properties, considerably alleviating incorrect immune responses and favoring osteogenesis [11]. The immune system plays a central role in coordinating the repair and regeneration of damaged tissue after infection or injury. In the inflammatory phase, the regulatory function of the immune response in the bone healing, repair, and regeneration induced by biomaterials has been demonstrated [12]. Biomaterial-induced immunomodulation can provide space for osteoblast growth and maturation during the repair and remodeling phase. Thus, the theory of osteoimmunology has eventually emerged.

The pivotal advantage of osteoimmunology is that we can grant bone material-specific biological properties to artificially modulate the local immune environment so that it inversely favors the process of osteointegration of the implant and osteogenesis [13]. To fulfill this key goal, there has been an impressive evolution in the design of principles and manufacturing criteria. To date, osteoimmunology has shown that immune cells actively participate in bone pathophysiology through the release of regulatory molecules (e.g., BMP2, BMP6, VEGF, OSM, and RANKL) to exert imperative effects on osteogenesis, and the dysfunction of these cells usually leads to an imbalance between osteoclasts and osteoblasts, which results in subsequent osteoarthritis, osteolysis, and osteoporosis. Chen et al. first proposed the concept of osteoimmunomodulation (OIM, please refer to Table 1 for the abbreviations covered in this manuscript) with the interpretation of a novel favorable property of bone biomaterials to induce a beneficial immune environment for osteogenesis [14]. In the present review, we discuss the research actualities of osteogenesis and dominant bone biomaterials and their advantages and disadvantages. We will also introduce the latest achievements in the field of osteoimmunology and OIM to provide novel insights into the design and production of bone biomaterials.

## 2. Bone and Bone Cells

Similar to many other connective tissues in the body, bone tissues are composed of three basic components: cells, fibers, and matrix. However, the most notable feature of bone is the deposition of a large amount of calcium salt in the cellular matrix, which grants it solidness to form the skeletal system in the body [15]. Four types of differentiated bone cells exist: osteoblasts, osteocytes, bone lining cells, and osteoclasts. They are responsible for the formation, composition, and degradation of bones. Notably, osteoblasts and osteoclasts are crucial for bone modeling and remodeling in the bone microenvironment, which is pivotal for maintaining bone homeostasis [16].

Modeling and remodeling occur constantly in bone tissues, which correspond to the decomposition and absorption of decrepit bone substance and the formation of new bone substance [17,18]. Osteoclasts are responsible for the removal of mineralized bone, and osteoblasts are responsible for the formation of the bone matrix and mineralization. There are three consecutive phases in the remodeling cycle: resorption, reversal, and formation. In the resorption phase, hematopoietic cells migrate to remodeling sites, where they differentiate into mature osteoclasts to digest old bones. The quiescent bone surface is covered by bone lining cells. In the reversal phase, when monocytes appear on the bone surface, they couple bone resorption to bone formation by generating an osteogenic environment at the remodeling sites. In the formation phase, mature osteoblasts predominate and constantly form new bone [19,20]. Bone modeling is one of the most important events in the skeletal system because it is responsible for altering and adjusting bone structure to meet changing mechanical needs, as well as assisting with the repair of microdamage in the bone matrix to ensure timely bone metabolism and to prevent severe pathological problems [21].

Osteoblasts are responsible for the synthesis, secretion, and mineralization of the bone matrix [22]. Bone formation starts with the maturation of osteoblasts. During preosteoblasts differentiation, they gradually transform from mesenchymal stem cells in the bone marrow (BMSCs) to mature osteoblasts [23,24]. Runx2 of the runt-related transcription factor (RUNX) family is the central transcriptional factor responsible for regulating the process of cellular differentiation in MSCs [25]. To exert transcriptional effects on downstream genes, Runx2 tends to heterodimerize with Cbfa1, granting itself a greater DNA-binding ability and stability [26]. The dominant role of Runx2 can generally be described as inducing the commitment of MSCs to the osteogenic lineage, promoting the proliferation of osteoblast progenitors, and facilitating bone mineralization by stimulating osteoblast differentiation. Runx2 has been confirmed to be solidly expressed in free MSCs, and an increasingly elevated expression of Runx2 has been observed alongside the osteogenic lineage. Specifically, Runx2 expression is the highest in immature osteoblasts, second highest in preosteoblasts, and lowest in mature osteoblasts [27,28]. This expression pattern could be considered an indication of Runx2 functioning in osteoblast differentiation and generation. In addition to Runx2, several downstream genes play vital roles in osteoblast differentiation. Osterix (Osx), identified as a novel zinc finger-containing transcription factor, is an indispensable molecule that participates in bone formation. Unlike Runx2, Osx displays a consistent expression pattern in all developing stages of bone formation, and no cortical bone or bone trabecula can be formed in the absence of Osx. This suggests that Osx is essential for osteogenesis. Moreover, it has also been shown that Osx potentially acts as a downstream target of Runx2 because Runx2/Cbfa1 expression is not affected by Osx, whereas Runx2/Cbfa1 is necessary for Osx expression in osteogenic cells [29]. Furthermore, Runx2 actively interacts with other factors to upregulate associated osteoblast differentiation markers such as alkaline phosphatase (ALP) and osteocalcin (Ocn). Runx2 functions not only in the process of osteoblast differentiation but also in bone formation-related events. Type I collagen is the main component of bone tissue, and the two chains constituting this protein are encoded by Col1a1 and Col1a2. On the one hand, it has been validated that Col1a1 is transcriptionally activated by Runx2 [30]. In contrast, Col1a1 expression has been reported to have no effect on Runx2 expression [31]. Therefore, further investigation is required to validate the association between Runx2 and collagen production. Once they grow into mature osteoblasts, the progenitors obtain the phenotype and morphology of osteoblasts, with an observed prominent structure of rough endoplasmic reticulum and active Golgi apparatus, and locate themselves on the surface of the bones.

Osteoclasts are multinucleated giant cells that incorporate several monocytes that differentiate from the monocyte-macrophage lineage of hematopoietic stem cells [32]. Proliferative monocytes/macrophages (i.e., preosteoclasts) enter the blood circulation under the chemotaxis of multiple chemical factors and fuse into multinucleated giant cells driven by various transcription factors, cytokines, and other signaling factors (e.g., such as macrophage colony stimulating factor (M-CSF) and the receptor activator of the nuclear factor kappa B (NF-κB) ligand (RANKL)), eventually growing into osteoclasts. Osteoclasts are well known for their major functions in bone resorption [33,34,35,36]. Their cytoplasm contains a well-developed endoplasmic reticulum and Golgi apparatus, as well as many actively operating mitochondria and lysosomes. Another hallmark of osteoclasts is the elevated expression of tartrate-resistant acid phosphatase, matrix metalloproteinases (MMPs), and cathepsin K in cells, which are helpful for breaking down organic matrix proteins to serve the function of bone absorption [37,38].

## 3. Immune Cells Regulation of Osteogenesis

In addition to MSCs, there are a large number of immune cells in bone marrow, such as B cells, T cells, monocytes, and macrophages, which account for approximately 20% of the total cells in bone marrow [39]. The immune system is a powerful and diversified defensive weapon used by higher organisms to protect themselves from foreign threats and to maintain physiological homeostasis. The primary role of the immune system is to fight infections, repair damaged tissue, and restore equilibrium in the body [40]. At the most fundamental level, the human immune system can be divided into two interrelated branches: the innate and adaptive immune systems. The innate immune system is the initial line of protection for the body and is capable of producing a non-specific immune response without prior programming when in contact with a foreign material or wounded tissue [41,42]. Unlike innate immunity, the adaptive immune system is composed of lymphocytes (B and T cells) that can recognize specific antigens. After an initial contact, the antibody is programmed to react uniquely to the antigen. This process slows down the adaptive immune system in comparison to the innate immune system, but it increases its precision and creates a crucial “immunological memory” by storing early antigens for years [43].

### 3.1. Macrophage Responses in Bone Regeneration

**Figure 1:**
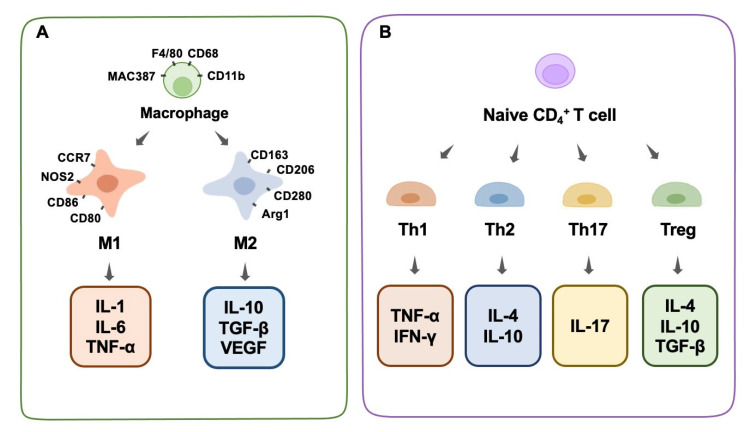
Macrophage phenotype and T-cell differentiation. (**A**) Macrophage phenotype, surface antibodies, and secreted cytokines. (**B**) T-cell differentiation and secreted cytokines.

Immune cells play a significant role in bone physiology and diseases by producing regulatory chemicals that influence osteogenesis. Macrophages are among the most important immune cells [44]. They play a crucial role in the immunological and inflammatory responses induced by biomaterials in the long term. They react to the debris of dead cells as well as external infections, which prioritize phagocytosis in the immune response [45]. It is well-known that macrophages possess a high-level intrinsic plasticity and a flexible polarizable activity into M1 and M2 subtypes (Figure 1A). These two subtypes of macrophages are classically distinguished based on diverse functional features, surface markers, and inducers [46,47]. Generally, well-recognized surface markers of M1 macrophages include CCR7, CXCL9, 10, and 11, CD86, NOS2, and others, and for the M2 phenotype include CD206, CD163, CD280, Dectin-1, Arg1, and others [48,49]. Functionally, macrophages play an indispensable role in the innate immune response of the human body, which is a pivotal part of the host defense. Despite the vague boundary of identification between M1 and M2 macrophages owing to the continuous properties of certain macrophages, these two subtypes could exert distinct effects on the processes of inflammation and the immune response [50].

Briefly, M1 macrophages are favorable for inflammatory responses and cytotoxic events in inflammation (designated as pro-inflammatory macrophages), whereas M2 macrophages tend to suppress inflammatory reactions and promote tissue repair (designated as anti-inflammatory macrophages). Classically activated inflammatory macrophages (M1) are recruited rapidly after tissue injury and participate in the early immune response, where they phagocytize pathogens and foreign infectious substances at the wound site. When M1 macrophages encounter infections, they secrete chemokines that attract other immune system cells (such as CD4+ T cells, CD8+ T cells, and dendritic cells), which transform structures from consumed cells to lymphocytes and other mature immune cells (antigen presentation) to cooperate in the phagocytosis process [51]. In addition, M1 promotes inflammation by secreting several cytokines, such as IL-1, IL-6, IL-17, IFN-γ, TNF-α, and TGF-β. In addition to serving as chemokines to boost the elimination of foreign matter during inflammation, these cytokines unintentionally harm normal tissues in several situations [52]. Due to the aggressive nature and fundamental function of the M1 phenotype in inflammation, it is impossible to ignore its important role in bone biology. Increasing evidence has shown that M1 macrophage-related inflammatory cytokines play a role in osteogenesis and bone-healing processes to some extent [53]. On the one hand, the various composition of inflammatory cytokines derived from M1 could have a diverse impact on the local osteogenesis process. Traditional recognition involves the collaboration of multiple chemokines, such as IL-17, IFN-γ, TNF-α, and TGF-β, which can induce the production of a mineralized matrix [54]. In contrast, TNF-α alone stimulates osteoclastogenesis and increases osteoclastic activity, resulting in bone resorption [55]. On the other hand, the partial enhancement of inflammation and osteogenetic reactions at a tissue level does not mean the enhancement of systemic bone formation at an organic level. In other words, the dual function of M1 macrophages in the immune response and inflammation has an undetermined role in osteogenesis and bone modeling within different situations. Generally, the robust pro-inflammation role of M1 macrophages decides the destroyed destiny of substantial bone tissues through a systematic reaction. Moreover, several recent studies hold the common view that, rather than M2 macrophages, moderately activated M1 macrophages are favorable for the osteogenic differentiation of mesenchymal stem cells (MSCs) through the mediation of OSM or bone morphogenetic protein 2 (BMP2) [56,57].

M2 macrophages, including the M2a, M2b, and M2c sub-categories, are generally considered to be responsible for tissue repair, in the form of a fibrocapsule or the formation of new bone, by alleviating the inflammation induced by the M1 phenotype [58]. M2a macrophages, produced by the activation of the cytokines IL-4 or IL-13, can suppress the secretion of several pro-inflammatory factors, such as IL-1β, IL-6, and TNF-α [58].M2b macrophages, also known as regulatory macrophages, secrete high levels of IL-10 and low levels of level of IL-12 [59]. M2c macrophages, produced by the activation of IL-10, glucocorticoids, or TGF-β, reduce the expression of various pro-inflammatory factors and enhance the ability to clear cellular debris [58]. The suppressive cytokines that M2 makes use of to resist inflammation mainly include IL-1RA, IL-10, and TGF-β, among which IL-10 and IL-1RA are known to promote osteogenesis [60]. IL-10 also inhibits osteoclast function [61]. In the repair process, M2 tends to increase multiple transforming growth factors (TGF-β1 and TGF-β3) to alleviate inflammation and facilitate fibrosis. Normally, this physiological process proceeds by the hallmark of fibrocapsule formation, which protects the normal tissue from the inflammatory microenvironment and ends in the hallmark of new bone construction [62]. It is worth mentioning that instead of a separate existence of the M1 or M2 phenotype, the macrophages continuously transform from the M1 stage to the M2 stage, and they successively perform specific duties in the whole wound-healing process. Therefore, the dynamic switching pattern highlights the significance of a proper switch from an M1 to an M2 phenotype during osteogenesis [63,64]. In other words, the appropriate proportion of the M1 and M2 stages of inflammation determines the outcome of bone regeneration. It could be concluded that any delayed switch from an M1 to an M2 phenotype would result in an inactivated M2 phenotype and poor tissue repair, and an early or excessive switch is bound to cause a deficient inflammatory reaction and delayed wound healing. Normally, the switch from the M1 to M2 phenotype could be recognized through the kinetics of gene level, surface marker expression, and protein secretion. The situation of the dominant M2 phenotype is associated with an increased expression of the M2 surface marker CD206, and a decreased expression of the M1 gene markers CCR7, IL-1β, and TNF-α, as well as an elevated level of M2-secreted proteins such as CCL18 and PDGF-BB [64].Based on a solid understanding of macrophage function, macrophage polarization and modulation could, therefore, be manipulated by designed biomaterials to fulfill specific bone-engineering events, such as osteogenesis and osteoclastogenesis [65].

### 3.2. T-Cell Responses in Bone Regeneration

The primary components of the adaptive immune system are lymphocytes, including B and T cells. Activated T cells can surface-express the receptor activator of nuclear factor ligand (RANKL) to stimulate osteoclast production and bone resorption. RANKL binds to the receptor activator of nuclear factor-κβ (RANK) on the surface of pro-osteoblasts, activating the RANKL/RANK signaling pathway and directly promoting osteoclast formation and differentiation through the RANKL/RANK/OPG response axis [66,67]. However, T cells can also release interferon-γ (IFN-γ) to prevent osteoclast formation, thereby interfering with TNF receptor-associated factor 6 (TRAF6), a crucial player of the RANK/RANKL signaling pathway, to block the activation of this signaling pathway and inhibit osteoclastogenesis [68]. Additionally, by interacting with bone marrow dendritic cells (DCs), CD4+ T cells are essential for the bone immune milieu by transforming into osteoclasts via the RANK/RANKL pathway [69].

Th1 cells secrete IFN-γ, IL-2, and TNF-α, which activate macrophages and promote inflammation. In contrast, Th2 cells generate IL-4, IL-10, and IL-13, which suppress macrophage function [70]. Studies have shown that some biological materials can enhance the production of cyclooxygenase-2 (COX-2) in the body after implantation, which in turn induces the release of prostaglandins (PGE2), which are chemicals that regulate inflammation [71]. PGE2 limits the growth and activity of T helper 17 cells by blocking the synthesis of IL-12p70 (Th17). In contrast, PGE2 increases Th17 cell growth and IL-17 production by increasing IL-23 production, which ultimately results in inflammation and tissue damage [71]. In addition, research has shown that tissue-derived biomaterials can potently modulate the expression of Tbx21 and Th1 canonical genes while simultaneously enhancing Th2 expression, which is important in the restoration of functioning tissues [72]. Treg cells constitute less than 10% of peripheral CD4+ T cells. They inhibit a broad spectrum of immune cells and prevent excessive immunological responses. Treg cells can inhibit osteoclast formation by producing IL-4 and IL-10 [73]. We conclude that decreasing Th1/Th17 cells while increasing Th2 cells is beneficial for tissue repair and osteogenic differentiation. Furthermore, increased Treg cells can suppress osteoclastogenesis by secreting cytokines that impede osteoclast differentiation (Figure 1B).

The body’s immunological effects can be tuned in a complex and delicate balance by transforming particular CD4+ T cells (such as Th1, Th2, Treg, and Th17) into each other. Therefore, the utilization of biomaterials in regulating balance is crucial for demonstrating the efficacy of immunomodulatory treatments in wound-healing and bone-regeneration processes [72].

### 3.3. Other Immune Cells Responses in Bone Regeneration

Neutrophils, the initial line of defense of the innate immune system, are capable of rapidly recruiting at areas of infection or tissue damage to eliminate pathogens and clear away debris. Their overactivity, which is initiated by infection or damage, generates a large elevation of pro-inflammatory cytokines and ultimately results in tissue destruction [74]. However, a growing number of studies have indicated that neutrophils perform additional functions. They can actively coordinate the regression of inflammation and help in tissue repair by interacting with cells of the innate and adaptive immune systems to modulate the immune response [75,76]. It was shown that IL-8, which is usually regarded as the most potent neutrophil chemotactic factor [77], is secreted at the location of bone abnormalities after bone injury. Neutrophils initially arrive at the site of the defect and recruit BMSCs and macrophages. Macrophages then regulate the differentiation of BMSCs towards chondrogenesis and osteogenesis [76]. At various stages of bone regeneration, neutrophils resemble macrophages and can polarize into N1 (pro-inflammatory) or N2 (anti-inflammatory) phenotypes at different periods of the inflammatory milieu [78] and then mediate immune responses or tissue healing accordingly. In the early stage of inflammation, high levels of IL-8 produce a pro-inflammatory milieu in which neutrophils are the pro-inflammatory N1 subtype. N1 recruits other types of immune cells (such as M1 macrophages, Th1 cells, and Th17 cells), aggressively reduces inflammation, and prepares the milieu for bone regeneration. After the inflammation subsides, IL-8 levels decrease and the neutrophils recruited during this phase are the N2 subtype that express anti-inflammatory factors which facilitate bone regeneration. Kovtun et al. found that the removal of neutrophils led to hampered healing after fracture in a mouse fracture model [79].

B lymphocytes (B cells) regulate bone formation and have a significant role in the risk of bone metabolism disruption [80]. B lymphocytes’ impacts on bone cells are mediated by cytokines and molecular pathways that have important effects on both immune cells and bone cell function, such as the RANKL/RANK/OPG signaling pathways [81,82]. Under normal circumstances, B cell-derived RANKL is required for B cells development; nonetheless, the overexpression of RANKL by activated B lymphocytes can have major consequences for bone metabolism [83,84,85]. In addition to RANKL/OPG, B cells can regulate bone homeostasis by generating several cytokines and chemokines, including TNF-α, TNF-β, IL-6, IL-10, and CCL3, which can modulate bone modeling and bone remodeling by acting directly on bone cells and modulating the immune microenvironment [86,87]. LPS-treated B lymphocytes have been shown to suppress the osteogenic function of rat bone marrow stromal cells via activating the Notch signaling system [88]. Furthermore, B cells have been shown to be able to differentiate into osteoblasts in vitro in the presence of 1,25(OH)2 vitamin D3 and ST2 stromal cells, or M-CSF and RANKL, thereby regulating bone metabolic processes [89,90,91].

### 3.4. Synergetic Regulation of Immune Cells in Bone Regeneration

**Figure 2:**
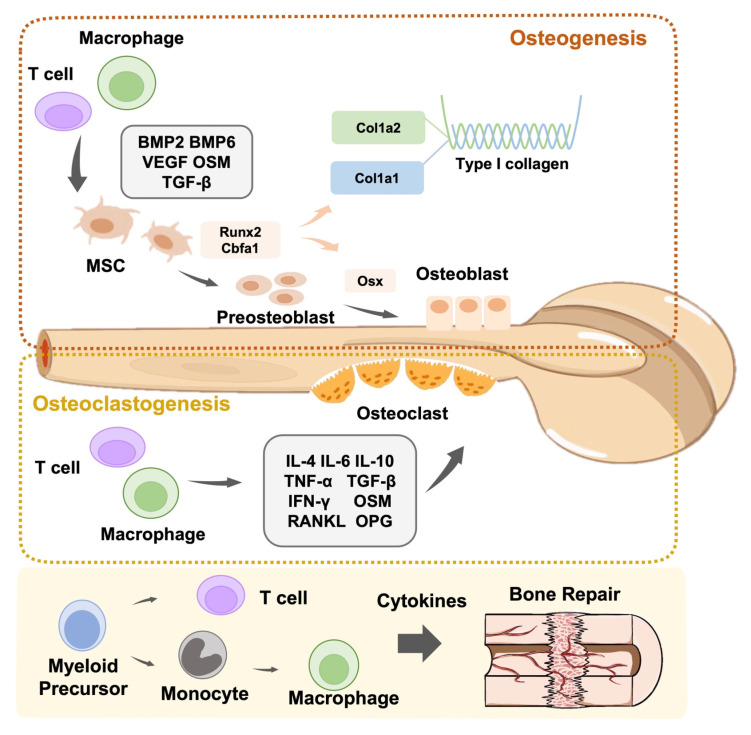
Schematic representation of the role of immune cells in bone modeling and remodeling. Immune cells are actively involved in osteoclastogenesis and osteogenesis.

Macrophages are the first biological response to allogeneic biomaterials. These extremely flexible immune sentinels govern and modulate the response to foreign and natural elements [92]. The destruction of the innate immune defense line causes macrophages to present antigen information to T cells through adaptive immunity, leading to the differentiation of T cells into Th1 and Th17 cells. IL-1, IL-6, and TNF-α are secreted by M1 leading to local inflammation. This stimulates Th1 cells to release pro-inflammatory cytokines (e.g., TNF-α, TNF-β, and IFN-γ), leading to a Th1-type inflammatory response. M2 macrophages secrete VEGF and TGF-β to help Th2 cells release cytokines such as IL-4, IL-6, IL-10, and IL-13, resulting in the generation of Th2 cells with anti-inflammatory properties that aid in tissue healing [93]. Conversely, T cells are also required for the functional polarization of M0 macrophages to the pro-inflammatory M1 or anti-inflammatory M2 type [94]. In addition, neutrophils recruit Th17 cells that can release the pro-inflammatory factor IL-17 to inflammatory areas by releasing CCL2 and CCL20 [95]. In turn, the pro-inflammatory cytokine IL-17 encourages epithelial cells to release CXC chemokines, hence enhancing neutrophil recruitment and activation [96]. In summary, when employing biomaterials to repair damaged tissues, it is critical to regulate the immune response and reveal the activation process of immune cells, notably the interplay between T cells and macrophages (Figure 2).

## 4. Biomaterial-Mediated Bone Regeneration Immune Response

The research and development of traditional osteogenic materials largely focuses on the direct filling of bone-defect areas based on the principles of mechanical physics and chemistry; that is, they basically restore the defect in appearance, provide good mechanical support, and have a chemical composition similar to that of natural bone tissue. This approach ignores the fact that bone-defect repair is a dynamic physiological process that involves a variety of cells and cytokines. Implanting bone substitutes invariably alters the entire bone microenvironment. Osteogenic differentiation is regulated by the new bone microenvironment formed by bone substitute materials and multi-system cells, rather than by the materials acting alone. The developed bone-substitute materials may improperly regulate the microenvironment, resulting in bone regeneration failure in vivo by disregarding the importance of other system cells and their microenvironment.

**Figure 3:**
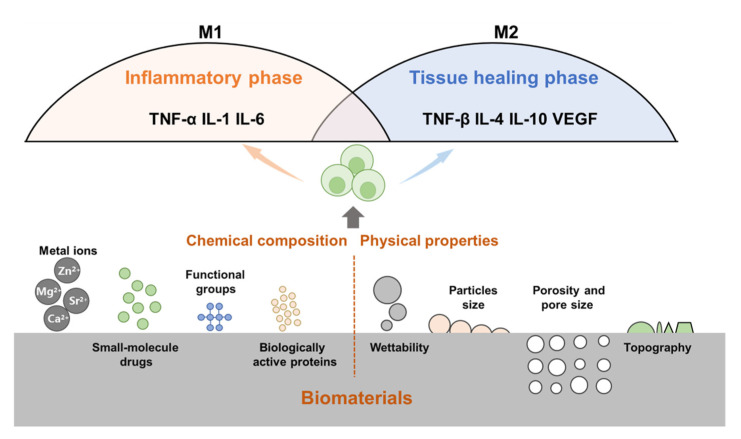
The physicochemical properties of bone biomaterials influence the immune response. For example, wettability, topography, particle size, porosity and pore size, release of metal ions, small molecule drugs, active proteins, and surface functional groups can modulate immune cells (e.g., macrophages) and their immune responses.

Following the implantation of biomaterials into the body, the immune cells of the body will respond immediately, identify allogeneic biomaterials, and initiate host defense responses. Research has shown that bone repair can be regulated by changing the chemical composition (metal ions, proteins, and small-molecule drugs) and physical properties of biomaterials (particle size, porosity, pore size, and topology) (Figure 3).

### 4.1. Chemical Composition of Osteogenic Biomaterials

Bone biomaterials typically degrade to varying degrees after implantation, releasing metal ions that alter the local microenvironment and impair the bone-bonding capability of the materials [97]. Metal ions can exert a crucial influence on immune and inflammatory responses through the direct regulation of macrophages, which is also considered to be an indispensable pathway [98].

Calcium (Ca) is a key component of calcium phosphate, a popular bone graft substitute that has been confirmed to connect with inflammatory signaling pathways [99]. High levels of extracellular Ca^2+^ have been reported to boost the production of Wnt5A by activating calcium-sensing receptor (CaSR) signaling, which could downregulate TNF-α and help reduce inflammatory reactions [100]. However, numerous inflammatory cytokines (e.g., IL-6, IL-1, TNF-α) can trigger bone resorption by stimulating the ligand of the receptor activator for NF-κB in osteoclasts [101]. Recently, it was found that certain cytokines such as IL-6 and IL-1 can upregulate the level of parathyroid CaSR, leading to hypocalcemia and the accumulation of phosphate, which inhibits bone resorption [102]. Hydroxyapatite is another commonly utilized bone biomaterial that promotes bone formation by stimulating osteoblast differentiation via the BMP2 and Wnt signaling pathways [103,104]. Therefore, the mutual relationship between calcium and inflammatory cytokines in the regulation of microenvironment inflammation and bone generation requires further investigation.

Magnesium (Mg), a mechanically bone-like metal ion, has a considerable biodegradability and biocompatibility in vivo and has been widely used in orthopedic implants [105]. In terms of inflammation, Mg can prevent the generation of inflammation-related cytokines by inhibiting the toll-like receptor (TLR) pathway, through which macrophages recognize foreign bodies and facilitate the innate immune reaction to deal with bone biomaterials [106]. Extracellular Mg^2+^ is reportedly an immunomodulator that modulates T-cell activation by binding to LFA-1 MIDAS [107]. It has been observed that memory T lymphocytes exhibit an Mg^2+^-dependent dose response in the production of activation markers and cell clustering [108]. In terms of bone modulation, it has been reported that high levels of Mg^2+^ could suppress osteoclastogenesis and reduce bone resorption. The experimental validation of RAW 264.7 cells cultured on the Mg-containing surface showed that these precursor cells failed to differentiate into mature osteoclasts [109]. These promotive properties for immune repression and bone formation make Mg an excellent metal ion dopant for bone biomaterials. Bioactive ions have numerous properties, as well as complicated biological connections with in vivo molecular processes, immune responses, and cellular compositions.

Strontium (Sr), a trace element, has been proven to be necessary for bone growth, promoting osteogenesis while suppressing osteoclastogenesis, and is extensively applied in the treatment of osteoporosis [110]. Sr-doped biomaterials have been proven to considerably promote early osseointegration [111]. Sr is highly dose dependent. Low concentrations of Sr are favorable for osteogenesis, whereas excessive quantities of Sr harm the surrounding microenvironment and cause apoptosis [112,113]. It has been reported that 250–500 μM Sr^2+^ causes the best osteoinduction [114]. Shen et al. successfully fabricated Sr-doped titanium surface coatings. In large-proportion strontium-doped materials (75–100%), the expression of anti-inflammatory cytokines (e.g., IL-10) and osteogenesis-related genes (e.g., TGF-β1) increases when compared to Ti (Sr content 0%), while the expression of inflammation-favoring genes (e.g., TNF-α) decreases significantly [115]. The high-Sr samples prominently encourage macrophage polarization from M0 to M2, creating a favorable milieu for regulating OIM [116]. Research shows that Sr^2+^ can bind to CaSR, because of its comparable characteristics to Ca^2+^, and stimulate bone formation through the MAPK/Erk 1/2 signaling pathway [117]. In addition, Sr promotes the osteogenic differentiation of mesenchymal stem cells via the Ras/MAPK signaling pathway [118].

Zinc (Zn), an essential component in maintaining the regular function of immune cells, is intimately associated with the growth and activity of macrophages [119]. Zinc homeostasis supports the differentiation of monocytes in blood into macrophages in infected tissue. Dubben et al. reported that decreased zinc levels in monocytes promoted their differentiation and increased their maturation [120]. However, excessively low concentrations of serum zinc have been shown to inhibit the growth of monocytes in the peripheral blood, and a concentration of 100 µM was suggested as the minimum concentration [121]. In addition, Brazão et al. found that zinc supplementation enormously increased the number of peritoneal macrophages, enhancing resistance to *Trypanosoma cruzi* infection [122]. In contrast, zinc determines the fate of macrophages by inducing regulated cell death in a concentration-dependent manner through different mechanisms [123]. It is worth emphasizing that both extremely high and low concentrations of zinc significantly trigger diverse forms of cell death. For example, in the macrophage RAW 264.7, a zinc oxide nanoparticle (ZnO) treatment that overloaded cells with zinc resulted in the necroptosis and apoptosis of macrophages in an Nrf2-independent manner [124]. Meanwhile, using the genetic loss of SLC39A10 to diminish the zinc level, Gao et al. found that macrophages could be induced by zinc depletion to apoptotic cell death mediated by the p-53 protein [125]. Therefore, it could be hypothesized that zinc induces programmed cell death in immune microenvironments in a concentration-dependent manner. Finally, zinc also plays an important role in the inflammatory functions of many immune cells. On one hand, zinc connects to macrophage functions by participating in TLR signaling [126]. A previous study found that a set of TLR (e.g., TLR1, TLR2, and TLR4) elicited the recruitment of macrophage phagosomes in mitochondria and enhanced their bactericidal activity [127]. TLR signaling is activated by the phosphorylation of interleukin-1 receptor-associated kinase 1 (IRAK1), whose degradation requires a certain level of zinc in vivo [128]. However, zinc deficiency is unfavorable for the regular biological processes of macrophages. It was found that long-term zinc insufficiency upregulated NLRP3 inflammasome production and activated IL-1β secretion by macrophages [129], whereas short-term zinc depletion inhibited inflammation by repressing caspase-1 activation, caspase-1 activation, and IL-1β secretion [130]. T cells are also highly vulnerable to the effects of Zn, notably on the balance of distinct T-cell subsets. The lack of Zn lowered the production of Th1 cytokines (IFN-γ, IL-2, and TNF-α), whereas it had little effect on Th2 responses (IL-4, IL-6, and IL-10), resulting in an imbalance between the Th1 and Th2 subpopulations [131]. In addition to its tight association with immune cells, Zn has a role in osteoblast and osteoclast differentiation. Multiple zinc-containing compounds have been added to bone biomaterial coatings to test their osteogenesis capacity. The upregulated abundance of differentiation markers of osteoblasts suggested that Zn may suppress the differentiation of osteoclasts while enhancing the maturation and mineralization of osteoblasts through involved immune responses [132]. Taken together, these findings suggest a multifaceted role of zinc ions in the regulation of macrophage-induced inflammation in a variety of ways, which inspired us to fully understand and better manipulate metal ions in the use of bone biomaterials. Therefore, metal ions are of great value to be fully understood and used in biomaterials. Modulation of the bone immunological microenvironment to stimulate osteogenesis by changing the concentration of different metal ions may become a significant development strategy for new bioactive bone materials.

In addition to metal ions, making use of bone material characteristics by the inclusion of biologically active proteins can also alter the ability of osteogenesis, with an emphasis on boosting M2 activation. Several signaling molecules, including oncostatin-M (OSM), prostaglandin E2 (PGE2), and BMP2, play a crucial role in the mechanism by which macrophages stimulate bone regeneration [53]. BMP2 is essential for the regulation of macrophage polarization and secretion. Based on a previous study, not only may BMP2 supplementation decrease the expression of inflammatory cytokines in M1 macrophages, it may also considerably increase the number of M2 macrophages [132]. BMP2-modified calcium phosphate cement (BMP2-CPC) causes an increase in M2 macrophages, which secrete TGF-1 and IL-10 to promote the in vitro osteogenic differentiation of MSCs [133]. In addition, it has been reported that the interaction of integrin β1 with fibronectin increases the expression of PI3 kinase signaling and promotes the phenotype of anti-inflammatory M2 macrophages, whereas blocking this mechanism induces M1 macrophages. Integrin β1 with fibrinogen on hydrophobic surfaces results in the generation of M1 macrophages, most likely through NF-κB activation [134].

Biomaterial surfaces with soluble anti-inflammatory small-molecule drugs (such as dexamethasone) can diminish the inflammatory response and fibrous encapsulation formation [135]. However, these drugs have limited applications because of their complex pharmacokinetics and decreasing concentration over time. Furthermore, the effect of anti-inflammatory agent coatings on biomaterials to promote osteogenesis needs to be considered. Glucocorticoids have been proven to improve inflammation relief and tissue repair when combined with anti-inflammatory cytokines (e.g., IL-6 and IL-10). However, glucocorticoids may decrease endogenous angiogenesis and increase the risk of infection [136]. Burgess et al. addressed this issue by administering dexamethasone and vascular endothelial growth factor (VEGF) via a hydrogel with an anti-inflammatory effect without impairing the formation of new blood vessels [137]. In addition, osthole, a coumarin-like derivative derived from traditional herbal medicine, has been found to enhance osteogenic differentiation [138]. Osthole stimulates osteoblast differentiation by activating the Wnt/β-linked protein/Bmp2 signaling pathway [139]. It has been reported that osthole can reduce inflammation by suppressing NF-κB [140] and has a negative effect on osteoblastogenesis and bone resorption induced by the nuclear factor-κB ligand (RANKL) receptor activator [141]. Moreover, sodium butyrate, a fermentation product of gut microbiota, loaded onto bone implant materials has been reported to have superior antimicrobial and osteogenic properties [142]. Sodium butyrate enhances macrophage phagocytosis by increasing reactive oxygen species (ROS) production. Sodium butyrate-loaded biomaterials increase macrophage M2 conversion and the secretion of anti-inflammatory factors, ultimately boosting bone healing [142,143]. The loading of different cytokines and biomolecules onto implant materials generates new ideas for the development of novel osteogenic materials.

Changing the characteristics of adsorbed proteins with distinct functional groups on the surface of biomaterials can also alter macrophage responsiveness. Buck et al. investigated the influence of surface chemistry on the behavior of macrophages on poly(polystyrene) surfaces with shared functional groups and comparable surface densities. The COOH group underwent adhesion with a large number of integrin-related proteins prior to macrophage attachment and improved the secretion of anti-inflammatory cytokines. After 48 h of culture, macrophages showed a pro-secretory effect on proteins associated with inflammation relief and tissue repair. In addition, unlike the NH2 and PO3H2 groups, the COOH group attenuated the LPS-stimulated inflammatory response of macrophages [144]. In addition, numerous studies have demonstrated that the COOH group is a possible anti-inflammatory surface functional component. According to Visalakshan et al., a surface with COOH groups resulted in a greater amount of LPS-stimulated macrophage IL-10 production and a lower amount of inflammatory cytokine release compared to a surface with amine or methyl groups. They also found that a hydrophilic AC surface containing COOH functional groups had a great affinity for albumin, which initiates the M2 pathway by encouraging the secretion of anti-inflammatory cytokines while blocking the production of inflammatory cytokines [144]. Conversely, a hydrophobic surface containing CH3 functional groups was more likely to adhere to IgG2, which can increase the release of inflammatory cytokines and suppress the release of anti-inflammatory cytokines, thereby activating the M1 pathway [145].

### 4.2. Physical Properties of Osteogenic Biomaterials

The surfaces of osteogenic biomaterials are in direct contact and react with the surrounding immune environment. Immune cells in the surrounding environment are affected by wettability, porosity, pore size, particle size, and surface microstructure.

The surface wettability of biomaterials is closely related to the adsorption of proteins, formation of blood clots, and formation of fibrin [146]. Early in the process of the implantation of biomaterials, vascular injury may lead to the extravasation of blood around the implant, triggering blood–biomaterial interactions. Blood clots play a role in the pool of cytokines that initiate wound healing. Hydrophilic polymeric surfaces may reduce protein adsorption and leukocyte activation, resulting in a lower rejection of foreign bodies [147]. By increasing the hydrophilicity of the material, implants can be more effectively integrated into the bone. A hydrophobic surface, when compared to a hydrophilic one, is generally capable of improving monocyte attachment and triggering stronger local immune responses. According to a recent study, a hydrophilic/neutral copolymer surface can significantly inhibit the adhesion of monocytes/macrophages, with the fusion of macrophages being minimal or absent, which effectively reduces the levels of vital factors, such as IL-6, IL-1β, and TNF-α [148]. According to Zischke et al., a hydrophilic surface modified by titanium (Ti) exhibited more active osteoblast differentiation, increased growth factor production, and higher osteogenic gene levels than unmodified surfaces [149].

Macrophages are approximately 20 μm in length [150]. When designing bone biomaterials, the porosity and pore size of osteogenic biomaterials must be considered, as these parameters may influence osteoblast function, macrophage polarization, and immunological responses. Micropore-size-appropriate biomaterials can promote macrophage secretion of VEGF by establishing a slightly anoxic external microenvironment, which induces the formation of microvessels and promotes bone regeneration [151]. Pores that are too small hinder the blood from transporting nutrients and oxygen, which will enhance the local inflammatory response, resulting in the formation of granulation tissue and completely blocking the micropores. The blockage prevents bone cell ingrowth from taking place, ultimately resulting in poor bone regeneration and implant failure [152,153]. Biomaterials with pore sizes of 90–120 μm have been shown to promote chondrogenesis and inhibit vascularization. Biomaterials with pore sizes up to 350 μm promote osteogenesis and vascularization [154]. Furthermore, high porosity facilitates the adhesion and growth of osteoblasts [155], resulting in the formation of a dense extracellular matrix, thereby enhancing early biological fixation [156]. In addition to their significance in bone cell activity, porosity and pore size also play an important role in the communication between the implants and the internal immune system. It has been shown that foreign body response activity diminishes as the pore size increases [156]. The foreign body reaction mediates the fibrotic response to encapsulate and remove foreign bodies from the surrounding tissue [157]. During a foreign body reaction, macrophages generate TGF-β which plays an important role in modulating the fibrotic response [157].

The size of the particles has a significant impact on the immune response. Implant particles are degraded and processed by immune cells based on their size. Macrophages can directly phagocytose particles with a small diameter (less than 0.5 μm). Although individual macrophages are no longer capable of phagocytizing structures larger than 0.5 μm, a foreign body giant cell is created as a result of the fusion of multiple macrophages in an attempt to phagocytize the particles [158,159]. When the material is excessively large (more than 100 μm), it obstructs macrophage phagocytosis and fusion [160,161], resulting in a highly inflammatory environment in which macrophages produce a substantial amount of inflammatory cytokines and ROS to destroy the substance. However, that is not to say that larger particles elicit a higher immunological response. Smaller particles in comparable-quality materials have a larger surface area, which results in an increased chemical activity and a greater immunomodulatory capacity [162,163]. Laquerriere indicated that small-diameter hydroxyapatite particles can trigger immune cells to release increased amounts of pivotal cytokines favoring inflammation [164]. Davison et al. discovered the presence of more multinucleated osteoclast-like cells surrounding calcium phosphate bioceramic crystal particles with a size of 1 μm as compared with crystals of 2–4 μm. RAW 264.7, which adhered to crystal particles with a size of 2–4 μm, was dramatically inhibited in RANKL-induced proliferation and differentiation into multinucleated osteoclast-like cells [165]. Furthermore, Li et al. compared the difference in OIM between two calcium phosphate bioceramics with submicron/micron surface topography and found that submicron biomaterials can regulate macrophage polarization to M2 by activating the PI3K/Akt pathway in vitro, thereby promoting the osteogenic differentiation of MSCs [166].

Another important property that influences immune-cell interactions is the surface topography of biomaterials [167]. There is plenty of research showing that modifying the surface topography can successfully regulate osteoblastic cell adhesion, migration, proliferation, and differentiation [168,169]. Surface roughness is an important modulator of both osteoblastogenesis and osteoclastogenesis. Polishing and sandblasting are two methods for modifying surface roughness, which can typically be portrayed on a microscale. For example, the surface of bone biomaterials has a prominent property in that it elicits modulative effects on the immune response in the host, and as a commonly used metal ion on the surface, Ti plays a regulatory role in the immune response. The surface topography of biomaterials is normally determined by the roughness of Ti, which has been shown to affect cell adhesion and spreading. When the roughness of the Ti-composed surface increases, the spreading of macrophages also advances [170]. In addition, titanium roughness also exerts significant stimulatory effects on macrophages to modulate the generation of inflammatory cytokines and chemokines [171,172]. Compared to the smooth Ti substrate, which promoted M1 polarization, Hotchkiss found that microroughened Ti surfaces encouraged the M2 macrophage phenotypic switch and increased the production of IL-4 and IL-10 [173]. According to Christo et al., materials with a 68 nm controlled surface nanotopography result in an increased synthesis of matrix metalloproteinase-9 (MMP-9) and a decreased release of pro-inflammatory cytokine secretion from primary macrophages when compared to the smooth glass control [174].

## 5. Definition and Research Status of OIM

Bone biomaterials modulate the local immune microenvironment and influence bone cell function, thereby regulating bone regeneration and reconstruction. Traditional bone biomaterials are primarily developed to consider whether they may lead to immune rejection and final implant failure. However, the implantation of bone biomaterials alters the microenvironment surrounding the bone. This produces a new microenvironment formed by the interaction between the bone biomaterials and multi-system cells that regulates osteogenic differentiation, rather than the materials alone. Immune cells play a central role in the local bone microenvironment as they regulate a variety of processes involved in bone regeneration (such as osteogenic differentiation, osteoclastic differentiation, fibrosis, and vascularization) by regulating the expression of chemokines and inflammatory factors. OIM is a novel concept for evaluating bone biomaterials that incorporates the new properties of biomaterials, bone cells, and immune cells to aid in the development of biomaterials. It pays more attention to the influence of the immune environment generated by the interaction with biomaterials on the behavior of bone cells rather than immune rejection. In short, biomaterials with a high OIM can elicit an appropriate inflammatory response via local immunocytes by releasing factors that drive the differentiation of BMSCs, ultimately leading to successful osteogenesis.

### 5.1. Evaluation Methods of OIM

Since OIM involves interactions between bone cells, immunocytes, and biomaterials, all components should be considered in the evaluation system, which can be achieved by a co-culture system. Indirect co-culture with a conditioned medium is a popular method. First, immune cells are cultured on bone biomaterials to obtain the supernatant containing cytokines related to the immune response. After mixing the supernatant with fresh medium, osteoblasts are grown in the conditioned medium to determine whether they have an influence on osteogenesis or the osteoclast reaction. This procedure is simple and reproducible. Additionally, this approach can be used when immune and bone cells originate from distinct species. However, indirect co-culture with conditioned medium cannot fully replicate the situation in vivo because bone cells actively govern the immune response rather than playing a passive role in the interaction with immune cells.

Indirect co-culture employing a Boyden chamber can more accurately mimic the in vivo environment associated with bone cell–immune cell interactions. Immune cells are seeded in the upper compartment and allowed to migrate through the pores of the membrane into the lower compartment, where bone cells and biomaterials reside [175]. A small pore size (0.4 μm) can retain cells in the upper chamber while allowing released substances to flow freely. A large pore size permits cells to migrate and can be used to evaluate the impact of activated immune cells [14]. The Boyden chamber assay saves time because it takes only a few hours for cells to pass through the porous membrane in the Boyden chamber, which is substantially less time than that required for cells to complete the cell cycle [176].

### 5.2. OIM-Based Development of Bone Biomaterials

OIM highlights the modulation of the immunological milieu formed by biomaterials, which plays a key role in the process of osteogenesis and the regulation of osteoclastogenesis. Bone regeneration requires the prompt modulation and transformation of the immune microenvironment in the region of bone defects from pro-inflammatory to anti-inflammatory. Macrophages are involved in a variety of biological activities, including infection, repair, and regeneration, as well as tissue homeostasis. Following biomaterial implantation, macrophages are the primary first response of the body’s immune system. It is therefore important to transform M1 macrophages into M2 macrophages, which drive osteoblast development and bone production by secreting BMP-2, IL-10, and TGF-β. When developing materials, it is possible to modulate the immune response by altering the composition or structure of the materials: (i) changing the material particle size, (ii) optimizing the pore size and porosity, (iii) increasing the hydrophilicity of the materials, (iv) adding metal elements to materials, and (v) combining the use of small-molecule drugs [177]. The current focus in the design of bone biomaterials is on inhibiting M1 macrophages and increasing M2 macrophages, thus releasing cytokines with anti-inflammatory effects in order to control inflammation and ultimately promote osteogenesis. The excessive suppression of M1 macrophages, however, limits the body’s ability to clear bacteria, making infection unmanageable and thus leading to the failure of osteogenic material implantation. In summary, the synergy between these strategies should be considered and any potential detrimental effects must be avoided when designing biomaterials to effectively induce bone reconstruction.

## 6. Conclusions

The complex process of bone healing and regeneration requires the accurate regulation of a series of molecular signals. The development of bone materials must consider the ability of the immune system to regulate the local microenvironment and facilitate bone formation. Biomaterials with a great OIM can induce an immune environment favorable for bone formation. By modifying the physical and chemical properties of biomaterials, such as their surface roughness and wettability, implants can directly influence immune cells in vivo, creating an optimal immunological microenvironment to dynamically regulate osteogenesis. Utilizing the immune system to regulate the bone-repair process precisely remains a great challenge, although we have established that immune cells are the primary force behind bone repair. Further research is required to investigate the complex signaling pathways and potential molecular targets of biomaterials, the immune system, and the skeletal system. Furthermore, numerous studies of implants have focused on the investigation of macrophages in microenvironments. However, there is still a dearth of studies exploring the role of other immune cells, including T cells, B cells, and neutrophils, in affecting the properties and functions of bone biomaterials. We suggest that more research attention should be paid to a comprehensive exploration of immune cells and their relationship with novel bone biomaterials in the future.

## Figures and Tables

**Table 1:** Abbreviations in this review.

Abbreviation	Full Name
OIM	osteoimmunomodulation
BMSC	bone marrow mesenchymal stem cell
RUNX	runt-related transcription factor
Osx	osterix
ALP	alkaline phosphatase
Ocn	osteocalcin
M-CSF	macrophage colony stimulating factor
NF-κB	nuclear factor kappa B
RANKL M1 M2	receptor activator of nuclear factor kappa B (NF-κB) ligand classically activated macrophage alternatively activated macrophage
IL-1	interleukin 1
IL-4	interleukin 4
IL-6	interleukin 6
IL-10	interleukin 10
TNF-α	tumor necrosis factor α
TGF-β	transforming growth factor β
MMP	matrix metalloproteinases
BMP2	bone morphogenetic protein 2
IFN-γ	release interferon γ
TRAF6	TNF receptor-associated factor 6
DC	dendritic cell
COX-2	cyclooxygenase 2
Th1 cell	T helper 1 cell
Th2 cell	T helper 2 cell
Th17 cell	T helper 17 cell
Treg cell	regulatory T cells
CaSR	calcium sensing receptor
TLR	toll-like receptor
IRAK1	interleukin-1 receptor-associated kinase 1
OSM	oncostatin M
PGE2	prostaglandin E2
BMP2-CPC	BMP2-modified calcium phosphate cement
VEGF	vascular endothelial growth factor
ROS	reactive oxygen species

## Data Availability

Not applicable.
